# Suicide prevention in Brunei

**DOI:** 10.1192/bji.2019.37

**Published:** 2020-05

**Authors:** Hilda Ho

**Affiliations:** FRCPsych, Head of Psychiatric Services, Ministry of Health, Brunei Darussalam. Email: hilda.ho@moh.gov.bn

**Keywords:** Suicide, stigma and discrimination, mental health policy, psychiatry and law, psychosocial interventions

## Abstract

Brunei Darussalam is a small country in Southeast Asia with a conservative Islamic government and culture. Suicide is a criminal offence under the civil Penal Code and the newly implemented sharia Penal Code. Suicide and mental illness are associated with significant stigma. Public discussion about suicide and mental illness has been considered taboo for many years. Recently, increased rates of media-reported suicides caused significant public alarm. This paper describes the initial steps that have been taken and possible future strategies for suicide prevention. The complex issues affecting suicide prevention in the national context are discussed.

Brunei is a developing country that scores highly in economic, health and social indicators. It is a member of the Commonwealth and was a British protectorate until 1984. Of the population of 421 300, 65.8% belong to the predominantly Muslim Malay ethnic group, 10.2% identify as Chinese and 23.9% belong to ‘other’ ethnic groups made up of Indigenous tribes and foreign residents.^1^ The government system is a monarchy and the Sultan of Brunei is the head of government. Brunei is a member state of the World Health Organization (WHO) Western Pacific Region. Healthcare services are provided by the government free of charge to all citizens. Mental healthcare services have undergone a period of development over the past decade.^2^ New mental health legislation has been implemented to improve the treatment, care and welfare of people affected by mental disorders.^3^ However, significant challenges remain. Brunei has a religiously and culturally conservative society. Mental disorders are highly stigmatised and beliefs about spiritual ‘uncleanliness’ are prevalent. People avoid seeking help for mental health problems.^4^ There are indications that the economy is slowing and unemployment rates are rising. Within this context, the country recently experienced an increase in suicides reported in the local media.

## Mental health services in Brunei

Mental health treatment is provided free of charge to all citizens in government-funded primary and secondary healthcare facilities throughout the country. The main specialist psychiatric facility is on a community site near the capital city, which houses in-patient, out-patient and rehabilitation services. There are 10 psychiatric beds per 100 000 population. Routine waiting times for a psychiatric appointment are under 1 month. Emergency psychiatric assessments are available in accident and emergency departments and walk-in services. Brunei has 1.7 specialist psychiatrists per 100 000 population compared with 1.6 psychiatrists per 100 000 population in the Western Pacific region^5^ and 2.6 psychiatrists per 100 000 population in Singapore, the country with the highest rate of psychiatrists in the region.^6^ There is a post-graduate training programme available, which aims to increase the number of psychiatrists. There are 4.8 psychologists per 100 000 population working in the government sector. Private psychology services are also available. Selected antidepressant and antipsychotic medications, including oral and injectable atypical antipsychotics, are available in government pharmacies. A larger medication list is available in a psychiatric clinic within a local private hospital.

## The prevalence of mental disorders in Brunei

There have not been any epidemiological studies of mental disorders in Brunei. However, it is possible to estimate the treatment prevalence for mental disorders as an indication of the proportion of people receiving treatment from mental health services. The government has implemented a national electronic health records system for all government healthcare services since 2012. As the government is the main healthcare provider, these electronic records are likely to cover most of the population. Data for all healthcare consultations recorded from October 2018 to October 2019 was used to calculate the number of patients who were given a mental health diagnosis by a health practitioner, as an estimate of the 1-year treatment prevalence. The rates for schizophrenia, schizotypal and delusional disorder (ICD-10 codes F20–F29); mania and bipolar disorder (ICD-10 codes F31 and F32); and depression and other mood disorders (ICD-10 codes F32–F39) were 580.2, 134.8 and 265.9 per 100 000 population, respectively. These figures are likely to be overestimates given that they include diagnoses made in primary and secondary healthcare facilities. Nevertheless, they compare favourably with treatment prevalence rates reported by high-income countries.^5^

## Suicide as a public health problem

The WHO has identified suicide as a major public health problem. Suicide prevention is a target of the WHO 2013–2020 Mental Health Action Plan. Traditionally, suicide was seen as a mental health problem to be managed by clinical interventions such as treatment for depression. More recently, an evidence-based public health approach using population-based strategies has been suggested to be more effective.^7^ Reducing the risk for the whole population may have a greater effect, as suicide has been shown to be caused by the complex interaction of biological, social, cultural, economic, environmental, psychological and behavioural factors.^8^ It is not clear which strategies would be most effective within Brunei's national sociocultural and legal context.

## Suicide rates in Brunei

Suicide rates are susceptible to procedural and sociocultural practices.^8^ The WHO Western Pacific Region has reported an age-standardised suicide rate of 8.4 (9.5 for males, 7.5 for females) per 100 000 population.^5^ Unfortunately, research in Brunei is hampered by the strong cultural taboo associated with suicide and mental illness. There are multiple difficulties in ensuring adequate data collection. Suicide and attempted suicides are classified as criminal offences and often thought of as sinful. This makes the community reluctant to report deaths as suicides. Consent for post-mortem examinations are often declined by family members. However, post-mortems are required to investigate the sudden deaths of foreign nationals. There has only been one published study on suicides in the country.^9^ This retrospective study evaluated 124 suicides that were brought by the police to the state pathologist for post-mortem assessment over a 20-year period. Foreign citizens made up the majority. Hanging was the most common method.

Recently, Brunei experienced an apparent increase in completed suicides reported by the police in local media. There was considerable government and public interest. Data reported with permission from the Royal Brunei Police Force showed an apparent increase in the national suicide rate from 1.9 per 100 000 population in 2015 to 3.6 per 100 000 population in 2018. However because of the absence of a systematic suicide inquiry system, it is not clear if these figures represent the actual suicide rates in the country. Hanging was the predominant method, occurring in 41 out of 44 cases recorded between 2015 and 2018, and 30 (68%) cases were foreign citizens. This may be related to the presence of many foreign low-income workers in the country.

## Rapid first response: setting up a mental health crisis helpline

There was increased public discussion about mental health and suicide. Social media users started asking for a telephone helpline. Although many telephone helpline services are operated by non-governmental organisations internationally, Brunei does not have a developed system of non-governmental organisations to do this. The Ministry of Health leadership decided to set up a telephone helpline operated by health professionals as a first response, to be supported by a nationwide mental health policy. A team was sent to neighbouring Singapore to observe the mental health crisis helpline operated by the Institute of Mental Health. It was decided that this model would be a ‘good fit’ and that some people would prefer using a telephone helpline as an initial step into mental health services. The common aims of mental health crisis helplines are to provide confidential out-of-hours support, crisis resolution and specialist advice.^10^ This new service was developed with similar aims. After a period of staff training, preparation and publicity, it was launched as a pilot project in February 2019. It will be evaluated to examine service use and outcomes.

## Media reporting

News reports of suicides often published personal details, videos or photographs of dead victims and their surroundings. Unfortunately, these details were widely shared on social media. A multi-agency press conference chaired by the Ministry of Health was held in August 2018 and media organisations were advised to ensure responsible reporting, to respect victims and the bereaved and to reduce the risk of suicide contagion. Media reporting improved significantly after this. However, there are no existing regulations or laws to ensure responsible reporting or to discourage the dissemination of inappropriate material on social media.

## Developing an evidence-based suicide prevention plan

Suicide prevention is a complex, multifactorial issue that requires a coordinated strategy from across the government and community. In Brunei, suicide prevention training has been provided for some health professional groups. Community mental health promotion activities are regularly carried out. Initial work with criminal justice and other agencies has been encouraging. However a coordinated suicide prevention plan should be part of a national mental health policy that aims to improve population mental health. An evidence-based approach would provide a useful framework. Preparatory work should include stakeholder analysis and population needs assessment. Guidance could be taken from suicide prevention plans among the WHO Western-Pacific countries, where common themes have included ensuring accurate data collection, multi-sector engagement, improving legislation to protect vulnerable people, training front-line workers, improving the quality and accessibility of mental health treatment, reducing stigma, ensuring media reporting standards and introducing programmes for high-risk groups.^11^ De-criminalising suicide and changing the focus from punishment to treatment may reduce stigma and improve research accuracy.^12^ Mental health indicators can be used to monitor the population's state of mental health. These should cover demographic and socioeconomic factors, health status, determinants of health such as personal conditions or the environment, and health systems.^13^

## Challenges

People with mental health problems are often reluctant to speak out for fear of stigma and discrimination. High risk groups, such as the LGBT+ community, substance misusers, children in care and prisoners, can be difficult to access. There is no antidiscrimination legislation to ensure access to education and employment for people with mental disorders or special needs. There are no patient advocacy services. Thus, it may be difficult to conduct population needs assessments. The police are often involved in serious self-harming incidents that are reported in the community or that present to emergency services. This can make it intimidating for people to seek help and arguably worsens stigma and distress. Health services find themselves dealing with clients who have different interests; the patient and the criminal justice system. The criminal procedures relating to mentally disordered offenders are lengthy and may result in a long period of detention.^14^ Nevertheless, most suicide attempts are not pursued in court. Although hanging appears to be the most common method of suicide, other less obvious methods may be overlooked because of the absence of a systematic suicide inquiry system.

## Sustainability

Any meaningful change will require substantial community and individual engagement. Current efforts rely on already stretched government clinical services and personnel to design and implement strategies. However, most clinicians do not have specific training in conducting population-based research or in designing and implementing public policy. Significant investment will be required in community outreach, human resource, research and data systems, to ensure the sustainability of any policy.

## The way forward

Despite many challenges, Brunei has made some progress in suicide prevention. Establishing a telephone helpline service was a significant first step. The development of a sustainable national mental health policy should be a priority. Establishing a systematic suicide inquiry system would improve research accuracy. Legislative support would protect vulnerable people and ensure access to treatment. Ensuring the sustainable implementation of an effective suicide prevention plan will require national commitment.
